# NKB cells: A double-edged sword against inflammatory diseases

**DOI:** 10.3389/fimmu.2022.972435

**Published:** 2022-11-03

**Authors:** Nikunj Tandel, Sushmita Negi, Rajeev K. Tyagi

**Affiliations:** ^1^ Institute of Science, Nirma University, Ahmedabad, Gujarat, India; ^2^ Division of Cell Biology and Immunology, Biomedical Parasitology and Nano-immunology Lab, Council of Scientific and Industrial Research (CSIR)-Institute of Microbial Technology (IMTECH), Chandigarh, India

**Keywords:** innate lymphoid cells, inflammation, IFN-γ, NKB cells, Th1 cells, IL-18, IL-12, infectious diseases

## Abstract

Interferon-γ (IFN-γ)-producing natural killer (NK) cells and innate lymphoid cells (ILCs) activate the adaptive system’s B and T cells in response to pathogenic invasion; however, how these cells are activated during infections is not yet fully understood. In recent years, a new lymphocyte population referred to as “natural killer-like B (NKB) cells”, expressing the characteristic markers of innate NK cells and adaptive B cells, has been identified in both the spleen and mesenteric lymph nodes during infectious and inflammatory pathologies. NKB cells produce IL-18 and IL-12 cytokines during the early phases of microbial infection, differentiating them from conventional NK and B cells. Emerging evidence indicates that NKB cells play key roles in clearing microbial infections. In addition, NKB cells contribute to inflammatory responses during infectious and inflammatory diseases. Hence, the role of NKB cells in disease pathogenesis merits further study. An in-depth understanding of the phenotypic, effector, and functional properties of NKB cells may pave the way for the development of improved vaccines and therapeutics for infectious and inflammatory diseases.

## NKB cells and their immune functions

Technological advances in cell biology have allowed investigators to better understand the phenotypic and functional characteristics of individual immune system cell types. Further, *in vitro* and *in vivo* investigations have identified the contributions of various immune effector cells to infections and inflammatory diseases (1–4). Moreover, advancements in immunobiology have allowed increased understanding of the mechanisms that underlie the signaling pathways responsible for pathogen elimination (2, 5–10).

B and T cell populations in the adaptive immune system share functional similarities with cells in the innate immune system, including natural killer T (NKT) cells (11), γδT cells (12, 13), and B1 B cells (14–16). The latter cell subsets play regulatory roles by stimulating acquired and innate immune responses in the host in order to fight pathogens. Furthermore, the characteristic features of NK cells and ILC subsets have been well studied with respect to IFN-γ production in response to host cell invasion by infectious agents (17–20).When analyzing NK cells in the spleen and mesenteric lymph nodes (MLNs) of mice, a population of cells co-expressing the NK cell markers NK1.1 and NKp46, as well as the B cell markers CD19 and IgM, were identified (21). Distinct from conventional NK and B cells, these cells uniquely expressed CD106 and CD63, and lacked expressions of common lineage marker (CD3, CD4, CD8, CD11b, and CD11c). This novel cell population, with properties comparable to NK and B cells, was referred to as “natural killer-like B (NKB) cells” (CD19^+^NK1.1^+^) (21). Giemsa staining, electron microscopy, immunofluorescence staining, imaging flow cytometry, and immunohistochemistry investigations confirmed that NKB cells exhibit a morphology similar to lymphocytes. These cells contained a small amount of endoplasmic reticulum and lacked cytotoxic granules in their cytoplasm.

NKB cells are primarily localized to the marginal zone (MZ) of the spleen; CD19^+^NKp46^+^ NKB cells were observed in the human spleen (∽ 2.7%) as well as in the MLNs (∽ 2.3%) (21). Gene expression profile studies revealed that NKB cells predominantly express components of the B-cell receptor (BCR), members of the Ly49 family of NK cell receptors, the major histocompatibility complex-I and II (MHC-I and II), CD40, CD83, and a higher expression of IL-18 as well as the proliferation marker Ki67. NKB cells expressed elevated levels of the B cell transcription factor *Pax5* and low levels of the NK cell transcription factor *Id2*. These cells also expressed high levels of CD63 and CD106 but lacked expressions for several characteristic dendritic cell (DC) markers (CD11c), ILC markers (CD127 or IL-7Rα), and T cell markers (CD3). Studies involving several knock-out mouse models (*Rorc^-/-^*, *μMT* ^-/-^, *Id2* ^-/-^, *Rag1* ^-/-^, and *Il2g* ^-/-^) confirmed the roles played by the IL-2R common γ chain as well as *Rag* recombinase signaling in NKB cell development and/or maturation (21). Furthermore, common γ chain-associated cytokines (IL-2, IL-15, and IL-4) were required for NKB cell expansion and longevity.

NKB cells were unable to secrete two major effector cytokines, namely, IFN-γ and TNF-α, and did not exhibit NK-like cytotoxic activity. Analysis of the complementarity-determining region 3 (CDR3), the most hypervariable region of the BCR and TCR, revealed a non-Gaussian distribution in the length of CDR3 sequences and a restricted BCR repertoire in NKB cells. However, conventional B cells exhibited a Gaussian distribution of CDR3 sequences with a broad BCR repertoire. These findings suggest the distinct nature of NKB cells as compared with B and NK cells. NKB cells were phenotypically characterized following the microbial infection of mice. The NKB cells expanded for up to 24 h post-infection and these cells secreted a variety of cytokines (IL-6, IL-12, IL-15, IL-1β, and IL-18), suggesting important functions. Moreover, infection progression led to a gradual increase in IL-18 production, whereas higher levels of IL-12 production were observed during the early phases of infection. When exposed to microbial agents *in vitro*, NKB cells were able to transactivate Th1 cells and ILCs to produce IFN-γ. NKB cells that were adoptively transferred into *Rag1* ^-/-^ mice became activated, and expanded in response to *Listeria monocytogenes* infection, suggesting immune activation independent of conventional B and T cells. Furthermore, IL-18 appeared to be the signature cytokine produced by NKB cells (21–23). Co-culture assays demonstrated the rapid activation of both NK cells and ILCs that secrete IL-18 and IL-12 to clear bacterial infections.

The developmental origin of NKB cells was revealed by transferring either NK cell progenitors (NKP) or pro-B cells to B cell-deficient μMT mice. The transferred pro-B cells differentiated into NKB and B cells, whereas the NKP cells did not further differentiate. The Lin^-^CD122^+^CD19^+^NK1.1^+^ cell lineage facilitated the conversion of NKP cells to NK cells *via* IL-15 (binds with CD122), and the former cells further differentiated into Lin^-^CD122^-^CD19^+^NK1.1^+^ NKB cells. Hence, the subpopulation of Lin^-^CD122^+^CD19^+^NK1.1^+^ cells was termed NKB precursor (NKBP) cells. This was further confirmed by transferring NKBP cells into NKB-deficient mice, after which NKBP cells differentiated into NKB cells. However, the transfer of different B cell populations into μMT mice failed to produce NKB cells. In short, the Lin^-^CD122^+^CD19^+^NK1.1^+^ lineage was proposed as the relevant precursor to NKB cells. These cells exhibit the *bonafide* NKB cell development pattern as well as their functional responses to microbial infection (21).

The frequencies, locations of tissue residence, and phenotypic markers of NKB cells were defined using genetic murine models. The majority of IgM^+^ cells failed to display stains for other markers of NK cells. NK1.1^+^NKP46^-^CD19^+^ and NK1.1^-^NKp46^+^CD19^+^ cells resembled conventional B cells rather than the distinct NKB cell population (24). Sorted NK1.1^+^NKP46^-^CD19^+^ cells that were stimulated with lipopolysaccharide (LPS) for three days rapidly differentiated into CD138^+^Blimp-1^+^ plasmablasts. Further, the proliferation and survivability of splenic NK1.1^+^NKP46^-^CD19^+^ cells were assessed in the presence of *bonafide* NK cell homeostasis factors. The majority of the NK1.1^+^NKP46^-^CD19^+^ cells did not survive in the presence of IL-15, and several cells exhibited an NK1.1^+^NKp46^+^ phenotype. Additionally, anti-NK1.1 and -NKp46 monoclonal antibodies were bound to the relevant antigens present on BCR^+^ cell subsets. Nevertheless, it was suggested that the novel NKB cell population is part of the conventional B cell lineage, rather than a distinct population. Although the possible role of NKB cells in the pathogenesis of infectious and inflammatory disorders has been explored, the precise identity of this cell type requires further validation using antibody-based identification methods (24).

Kerdiles et al. utilized the *Ncr1*-driven *Cre* model, where inefficient expression of *Cre* may be a possible explanation for the low or absent expression of NKp46. Furthermore, Wang and colleagues reported that bone marrow-derived pro-B cells were the primary source of NKB cells that originated from NKBP cells. The transcription factors involved (as stated earlier for NKB cells), and the regulatory mechanisms by which pro-B cells are converted to NKBPs (the results of an adoptive transfer experiment carried out on μMT mice), followed by their maturation into NKB cells is of particular interest. Moreover, LPS stimulation failed to induce the production of IgM by NKB cells. These conflicting results may be attributed to the detection method employed by Kerdiles et al. Finally, NKB cells are distinct from conventional B cells and thus represent a unique subset of innate B cells that may play an important role during microbial infection. The conversion of pro-B cells to mature NKB cells requires further investigation (25).

## NKB cells and their role in infectious and inflammatory diseases

### NKB cell studies in SIV-infected non-human primates

**Figure 1:**
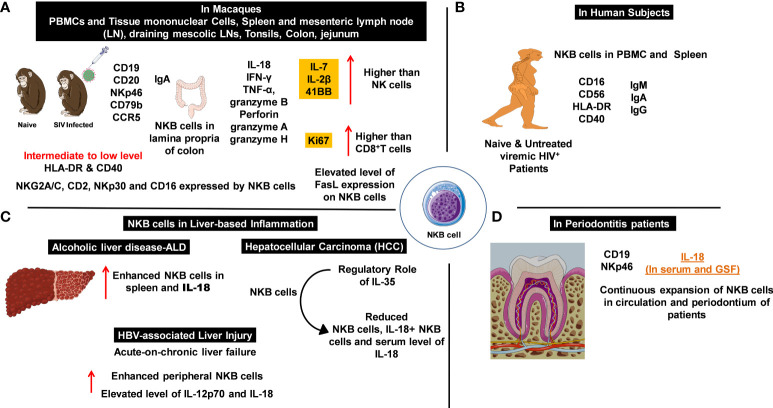
Natural killer-like B cells (NKBs) play a crucial role in the pathogenesis of infectious and inflammatory diseases. The expression profile of NKB cells with cytolytic granules and other signaling and defense molecules which help to understand inflammatory disorders. **(A)** NKB cells and their expression profile in the periphery and deep-seated secondary lymphoid organs of naïve and simian immunodeficiency virus (SIV)-infected macaques. Expression of inflammation mediators and cytolytic granules in cytotoxic immune cells. **(B)** The numbers and characteristics of NKB cells that were studied in the circulation and lymphoid organs of patients with viral infections. **(C)** The increased number of NKB cells is proportional to the elevated expression of IL-18 during inflammation observed in liver pathologies. **(D)** An increase in NKB cells mediates enhanced IL-18 production in patients with periodontitis.

Based on the discovery of novel NKB cell populations in rodents, Manickam et al. investigated NKB cells in macaques and humans (26) (**Figures 1A, B**). The peripheral blood mononuclear cells (PBMCs) and tissue mononuclear cells from various organs of uninfected and HIV-infected humans, as well as those from uninfected and simian immunodeficiency virus (SIV)-infected macaques, were analyzed. Similar NKB cell frequencies were identified in both groups of primates, and their enrichment in the spleen validated earlier findings carried out in rodents (**Figures 1A, B**). A broad distribution was also noted in other human organs such as the tonsils, colon, jejunum, and lymph nodes (LN). These cells expressed NKp46, moderate levels of CD16, and low levels of both HLA-DR and CD40. Molecular studies confirmed the expression of several NK cell markers (CD2, NKp30, NKG2A/C, and CD16) by NKB cells in both species (**Figures 1A, B**) (26).

Manickam and colleagues identified this unique NKB cell population in both naïve and chronically SIV-infected macaques. The distributions of NKB cells in different deep-seated organs varied in the uninfected macaques. NKB cells were differentiated from other cell types depending on their CD3^-^NKG2A^+/-^NKp46^+/-^CD20^+^CD127^-^ phenotype, as characterized by fluorescence-activated cell sorting (FACS). In healthy humans, NKB cells expressed CD40 and HLA-DR, and the levels of these markers did not significantly differ in HIV-infected individuals following antiretroviral therapy (ART). The expression pattern of surface immunoglobulin differentiated NKB cells from other cell lineages. High expressions of IgM and IgA and a low expression of IgG were observed in uninfected macaques and humans (**Figure 1B**) (26). It was estimated that IgG expression levels were increased 10-fold in both the spleen and MLNs of SIV-infected macaques, compared with the levels of PBMC in HIV-infected patients (26).

The role of NKB cells in gut-associated inflammation during mucosal immune responses to SIV infection was investigated (27). NKB cells present in the lamina propria of SIV-infected rhesus or cynomolgus macaque colons were compared with uninfected controls. RNA sequencing and flow-cytometry analyses showed that the NKB cells display receptors, markers, and functions similar to NK and B cells (27). The NKB cells were the primary source of IL-18 production in the colon following SIV infection. This was confirmed by staining lamina propria lymphocytes with anti-IL-18 antibodies and NKB cell marker antibodies. *In vitro* studies confirmed that NKB cells were the “natural source” of IL-18. Additional findings showed that almost 68% of the NKB cells (from the colon tissues of six infected subjects) produced IL-18, whereas none of the B or NK cells produced IL-18. Since IL-18 and IL-1β are canonically produced in response to inflammasome activation, IL-1β was investigated. IL-1β was produced by NK and B cells; however, no production was observed by the NKB cells (27). Thus, the NKB cells were concluded to be the primary source of IL-18, whose expression is highly regulated by a non-canonical pathway that mounts inflammatory responses in the SIV-infected colon. Transcriptional analyses demonstrated that NKB cells possessed a unique transcriptome, compared with that of NK and B cells. The NKB cells expressed both MS4A1 (CD20) and NCR1 (NKp46), whereas the B and NK cells only expressed MS4A1 and NCR1, respectively.

NKB cells expressed increased levels of granzyme H transcripts compared with NK and B cells. In NK cells, the Fas ligand (FasL) targets and destroys virus-infected cells. An increased expression for FasL in NKB cells was observed during viral infection (27). Furthermore, over 84% of the NKB cells expressed FasL, compared with 10% and 14% of NK and CD8^+^T cells, respectively. Protein expression confirmed elevated IFN-γ production (46.7% of NKB cells vs. 7.95% of NK cells and 8.14% of CD8^+^ T cells) in the infected macaque colons. These findings suggest that NKB cells may have originated from NK cells that were extracted from the colon. However, these findings contradict earlier results which showed that NKB cells originated from pro-B cells. The immunoglobulin (Ig) loci in the NK cells did not undergo *de novo* V(D)J recombination since expressions for μ heavy as well as λ and κ light chains inside the cytoplasm were observed. In addition, over 40% of the NK cells (excluding the CD56^-^CD16^+^ phenotype) expressed intracellular IgM. Hence, other B-cell molecules began to appear during SIV infection. CD79b expression plays an important role in surface Ig expression, depending on its interaction with the Ig heavy chain and CD79 (28). Neither NK cells nor CD79b expressions were observed in the SIV-infected colon, despite the intracellular expression of IgM. The NKB cells expressed IgA, similar to gut B cells, except for the co-expression of CD56 and CD20. B cells produce IgA with J chains upon conversion to plasma cells, therefore, CD79b and Ig are crucial for NKB cell signaling pathways. The co-expression of CD79b and IgA may be important since CD79b acts as a signal transducer and Ig may trigger the production and activation of NKB cells. The interaction between CD79 and the Igα heavy chain ruled out the possibility of poly-IgA playing a role, resulting in the monomeric expression of IgA. The expression mechanism for Ig in NKB and NK cells present in infected colons, which can allow confirmation of whether the NKB cells originate from NK or pro-B cells, warrants further investigation (27). These findings suggest that the expression of CD79b in NKB cells functions differently to conventional B cells. Furthermore, affinity maturation in the germinal center (GC) and downregulation of CD79b are essential for the selection of B cells in GCs, followed by antigen presentation to T follicular helper cells.

The signature pro-inflammatory cytokine IL-18 is produced by NKB cells together with IFN-γ and TNF-α. The production of granzyme B and perforin confirmed the cytotoxic nature of NKB cells (27). Moreover, significantly increased production of granzyme A was found in NKB cells, compared with NK and CD8^+^T cells. In addition, two subpopulations of granzyme A-producing NKB cells were observed, although their prevalence and phenotypic and functional characterization requires further investigation. IL-18 binds to IL-18Rβ secreted by NKB cells and facilitates both IFN-γ and TNF-α production during the pathogenesis of infectious and inflammatory diseases. The expressions for IL-7, IL-2β, and 4-1BB compared with NK cells, the increased Ki67 expression (proliferation marker) compared with CD8^+^ T cells (**Figure 1A**), and RNA-sequencing analyses demonstrated the enhanced proliferation of NKB cells compared with NK and CD8^+^ T cells. In brief, these studies presented the presence of newly identified NKB cell populations in the colons of SIV-infected macaques and other deep-seated tissues. The phenotypic characteristics shared by these cells with NK and B cells, together with their functional properties and enhanced proliferative activity (compared with NK and CD8^+^ T cells) indicates their important role during infectious pathologies. The origin of NKB cells, their role in SIV pathogenesis, pathways for both cytokine production and cytolytic activity, and the role of FasL on the surface of these cells (for loss of CD4^+^ T cells – primarily Th17 cells) (**Figure 1A**) all require further study (27).

### NKB cells in liver pathologies

#### Role of NKB cells in alcoholic liver disease

The studies discussed above suggest that NKB cells play a role in microbial infection and inflammation. A recent study on alcohol-induced liver disease (ALD) and intestinal damage, based on a chronic-binge alcohol abuse model, explored the therapeutic mechanisms of pre-activated (with toll-like receptor 3; TLR3) bone marrow-derived mesenchymal stem cells (P-BMMSCs) (29). Interestingly, elevated numbers of NKB cells in the spleen and significantly higher IL-18 serum levels were observed in alcohol-treated mice. These cells were shown to activate NK cells and ILC1s, leading to the aggravation of ALD. This was followed by chronic inflammation and increased lipid deposition (**Figure 1C**). Treatment with BMMSCs resulted in a reduction of NKB cell numbers and serum IL-18 levels (29).This confirmed the pathogenic role of NKB cells in ALD, which may be relevant to other inflammatory diseases. Additionally, P-BMMSCs indicated the involvement of TLR ligands in the immunosuppressive activities of NKB cells. However, the mechanism of action and the immunosuppressive activity of NKB cells are not fully understood. Nevertheless, NKB cells may be used to target infectious and inflammatory diseases and to develop therapeutic interventions (**Figure 1**).

#### NKB cells in hepatocellular carcinoma

Hepatocellular carcinoma (HCC) is one of the leading causes of mortality in cancer patients. The reasons for an impaired immunological network in HCC are not fully understood (30). IL-35 has been studied for its immunosuppressive activity towards both the hepatitis B virus (HBV) and HBV-associated HCC (31). Upregulated/activated IL-35 restricts the anti-tumor activity of CD8^+^ T cells in the tumor microenvironment. Additionally, IL-35 produced by the regulatory T cells (T_reg_) drives T cell exhaustion in the tumor microenvironment, as indicated by the increased expression of inhibitory receptors (PD-1, TIM-3, and LAG-3) (32). These interactions regulate the activity of Th9 cells in HCC (33). Moreover, IL-35 also regulates the tumor microenvironment in conjunction with other negative regulators [IL-18-binding protein (IL-18BP-an antagonist of IL-18)] (34, 35). This suggests that IL-35 may play an immunomodulatory role for NKB cells in HCC. This hypothesis was tested by studying both the peripheral and liver infiltrating NKB cells obtained from HCC patients (30). The number of NKB cell (CD3^-^CD19^+^CD56^+^NKp46^+^) populations that produced IL-18 in the peripheral and liver infiltrating sites of HCC patients were downregulated.

IL-35, IL-12, and IL-18 serum levels were quantified, revealing increased IL-35 and reduced IL-18 levels in HCC patients and confirming the regulatory role of IL-35 (30). Additionally, IL-35 serum levels were well correlated with the frequencies of peripheral NKB cells, IL-18^+^ NKB cells, and IL-18 serum levels (**Figure 1C**). The effect of NKB cells on CD8^+^ T cells was revealed using co-culture experiments (autologous CD8^+^ T cells cultured with HepG2 cells in the presence/absence of NKB cells or recombinant human IL-18BP). The cytotoxic activity of CD8^+^ T cells in the control group was augmented, compared with the HCC patient group. Human IL-18BP directly suppressed the NKB cell-mediated cytotoxicity of CD8^+^ T cells. Elevated levels of IFN-γ and TNF-α were identified in the supernatant of CD8^+^ T cells in the control group. Hence, the NKB cells promoted the cytotoxic activity of CD8^+^ T cells *via* IL-18 signaling.

Intrahepatic lymphocytes (IHLs) were stimulated with IL-35 (1 ng/ml) for 24 h and reduced frequencies of NKB and IL-18^+^ NKB cells were observed in both patients and healthy controls. Reduced IL-18 supernatant levels confirmed these results, and no changes were observed in IL-18BP secretion (30).The continuous secretion of IL-18BP neutralizes IL-18, decreasing its levels. Therefore, the regulatory effect of IL-35 towards NKB cells does not appear to be associated with IL-18BP. In order to study the role of IL-35 in regulating NKB cell activity, autologous CD8^+^ T cells were co-cultured with HepG2 cells in the presence/absence of NKB cells for 12 h and the cytotoxicity of CD8^+^ T cells was determined. The results suggest that IL-35 plays an immunoregulatory role in the NKB cells, mediating CD8^+^ T cell cytotoxicity and inducing tumor progression. The observed immunosuppressive activity of IL-35 in HCC patients raises several questions for future studies. For example, a major focus of this study was to determine the role of IL-18 on NKB and CD8^+^ T cells present in HCC patients, with similar levels of IL-12 observed in both HCC patients and healthy controls. However, the presence and functional activities of IL-12-producing NKB cells in the livers of both healthy individuals and HCC patients requires further *in vitro* and *in vivo* investigation.

#### Role of NKB cells in hepatitis B virus associated liver injury

The hepatitis B virus (HBV) is one of the etiologic factors for chronic liver disease, which can lead to acute-on-chronic liver failure (ACLF). The change from low-grade inflammation to chronic, pathogenic HBV-associated ACLF was studied (36). The authors performed assays on HBV patients to evaluate the regulatory properties of NKB cells and to determine the roles of IL-12 and IL-18 in HBV-associated liver injury. A phenotypic characterization of different lymphocyte (B, T, NK, and NKB cells) populations was performed on blood collected from patients (36). The gating strategies utilized for B and T cells were CD3^-^CD19^+^ and CD3^+^CD19^-^, respectively, while the CD16^+^56^+^ gate within the double negative population of CD3^-^CD19^-^ cells was considered to be NK cells. NKB cells were gated and differentiated using the phenotypic markers for CD3^-^CD16^+^56^+^NKp46^+^CD19^+^ cells. The major lymphocyte populations (primarily T, B, and NK cells) were largely unaltered in all experimental groups. However, a significant difference was observed in the peripheral NKB cells of HBV-ACLF patients, compared with chronic hepatitis B (CHB) patients, asymptomatic HBV carriers (AsC), and healthy controls (HC). A significantly elevated level of IL-12p70 was observed in the plasma of HBV-ACLF and CHB patients, compared with the AsC and the HC, whereas IL-18 levels were slightly altered in only the HBV-ACLF patients (36). The frequency of NKB cells positively correlated with IL-18 levels, but did not correlate with IL-12 levels in the HBV-ACLF surviving patients. Following therapy, a significant reduction in the number of NKB cells was observed in HBV-ACLF patients. Surprisingly, there was neither a change in IL-12p70 and IL-18 plasma levels, nor a correlation between the frequency of NKB cells and the liver injury index of HBV-ACLF patients. Therefore, the role of NKB cells in HBV-ACLF pathogenesis was not fully established.

IL-12 and IL-18, the signature cytokines of NKB cells, were used to stimulate PBMCs that were isolated from HBV-ACLF patients. Previous findings on infectious diseases and cancer (36) provided evidence for the increased frequencies of T and NK cells at two (1 and 10 ng/ml) IL-12 concentrations. No differences in the B and NKB cell frequencies were observed between unstimulated and IL-12-stimulated PBMCs (at 1 and 10 ng/ml). Nevertheless, a noticeable increase in the frequency of NKB cells in HBV-ACLF patients upon stimulation with a high concentration of IL-18 (10 ng/ml) was reported, but no changes were observed at a low concentration (1 ng/ml). Minor changes were observed in the T, B, and NK cell lineages when PBMCs were stimulated with IL-18. IL-18 was continuously produced by the NKB cells, and IL-12 production was monitored during the early phases of microbial infection (21). The HBV-X protein induced IL-18 expression in the liver which incurred hepatic damage during HBV infection. IL-12 plays a vital role in promoting central memory CD8^+^ T cells by reversing the exhaustion of virus-specific CD8^+^ T cells. Furthermore, *in vitro* stimulation assays using IL-12 and IL-18 suggested that IL-18 plays a direct role (positive feedback) in the signalling mechanism of NKB cells.

IL-18R signalling primarily activates two major pathways (MyD88 and STAT/MAPK) *via* NF-κB (phosphorylation). Reduced amounts of phosphorylated NF-κB p65 were observed upon stimulation with 10 ng/ml IL-18, compared with stimulation using 1 ng/ml (36). IL-18 levels were significantly reduced in the supernatant of cells stimulated with 10 ng/ml, while only a slight reduction was observed when cells were stimulated with 1 ng/ml IL-18. Thus, a lower IL-18 concentration did not allow complete neutralization of IL-18 by IL-18BP, resulting in phosphorylation of NF-κB. This directly enhanced the production of NKB cells in HBV-ACLF. The signature cytokines IL-12 and IL-18, and their role in NKB cell activation during microbial infections require further investigation. Further studies that explore the impact of NKB cells on B and T cells in different pathogenic settings may be useful in developing next-generation therapeutics.

### Pathogenic role of NKB cells in periodontal infection

Detailed pathogen-host interaction studies are required to better understand the role of cytokines and chemokines in the progression of periodontitis (2). The accumulation of IL-18 has been reported in patients with acute and chronic periodontitis (**Figure 1D**) (37). IL-18 knock-out mice exhibited a loss in periodontal bone during periodontitis caused by *P. gingivalis* (38). The presence of NKB cells and the production of inflammatory cytokines (IL-18) have been reported in patients with periodontitis. The pathogenic nature of NKB cells was confirmed in the *P. gingivalis*-induced periodontitis murine model (37). CD3^-^CD19^+^NKp46^+^ NKB periodontium cells were found to be the major source of IL-18 production in both the serum and the gingival crevicular fluid (GSF) of periodontitis patients; this was not the case in healthy individuals. No physiological changes were observed in the periodontal ligaments upon stimulation with recombinant IL-18. The neutralization of IL-18 suppressed bone loss, the infiltration of non-immune cells, and cytokine production (37). The continuous expansion of NKB cells in both the circulation and the periodontium of patients and mice with periodontitis suggests that NKB cells play a pathogenic role in intraoral infection. Following periodontal therapy, periodontal-infiltrating NKB cells were not observed, raising the possibility of a distinct lineage of lymphocytes (37). Due to the small sample sizes and the unclear sources of IL-18 secretion in these studies, the potential of NKB cells as a therapeutic regimen for periodontitis remains to be established.

### Role of NKB cells in rheumatoid arthritis

Rheumatoid arthritis (RA) is a heterogeneous, systemic autoimmune disease, characterized by synovitis, progressive bone damage, loss of joint function, and extra-articular manifestations (39, 40). Genetic analyses in experimental animals with RA and clinical investigations have revealed the genetic and environmental risk factors associated with RA and the ultimate propagation of chronic inflammation. The unregulated production of inflammatory cytokines (IFN-γ, TNF-α, IL-1β, IL-6, IL-15, and IL-18) is responsible for various disease complications (41) as well as the disruption of immune homeostasis (42).

Our *in vitro* and *in vivo* findings (43, 44) suggest the therapeutic potential of combination therapy using the anti-inflammatory drugs aceclofenac (ACE) and methotrexate (MTX). The intravenous delivery of MTX *via* lipid-polymer hybrid nanoparticles (LPHNPs) (45, 46), together with the topical application of ACE using nanostructured lipid carriers (NLCs) (44, 47), suggests that the induction of apoptosis in proinflammatory RA cells was regulated by NF-κB and FOXO1 transcription factors. Therefore, MTX+ACE-loaded nanoscale carrier-based co-therapy approaches can modulate RA-induced inflammation and can induce apoptosis in pathogenic cells. Our group has been elucidating both the mechanism underlying RA pathogenesis, as well as the capability of MTX+ACE combination therapy to modulate RA-induced inflammation and to establish immune homeostasis by maintaining the immune cell Th1 phenotype.

**Figure 2:**
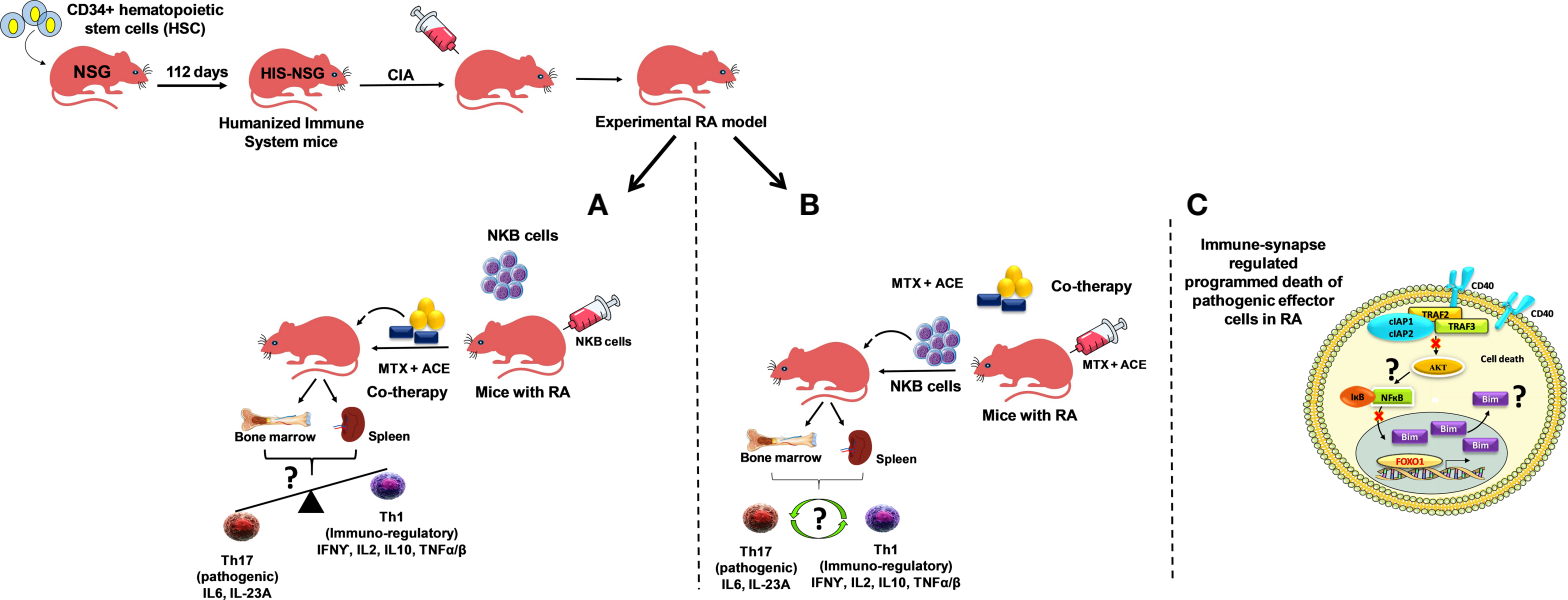
Experimental humanized immune system mice (NSG mice reconstituted with hematopoietic stem cells (HSCs); CD34^+^ cells repopulating the human immune effectors and referred to as HIS mice). Arthritis induced by collagen treatment (collagen-induced arthritis; CIA) in HIS mice (CIA-HIS) to study the role of combined therapy in the modulation of arthritis-induced inflammation. **(A)** CIA-HIS mice may be adoptively transferred with NKB cells followed by aceclofenac- and methotrexate-based combination therapy (co-therapy) to assess the immunoregulatory (Th1) phenotype in immune cells, allowing the progression of RA to be determined, and **(B)** co-therapy treatment in CIA-HIS mice followed by adoptive transfer of NKB cells. **(C)** CD40-mediated and NF-κB-controlled downstream effectors of the PI3K-Akt pathway (Akt1) and pro-apoptotic protein (Bim) induction during the signaling pathway to drive programmed death in pathogenic effector RA cells.

Collagen-induced arthritis in mice possessing a humanized immune system (CD34^+^ cells reconstituted immunodeficient (NSG) mice repopulated with a human immune system; HIS) may be employed to confirm the role of NKB cells during RA. NKB cells can be adoptively transferred to these CIA-HIS mice, followed by treatment with ACE+MTX (**Figure 2A**). This may subsequently be followed by immunophenotyping for Th17 (IL-6 and IL-23A) and Th1 (IFN-γ, IL-2, IL-10, and TNF-α/β) marker expressions using cells extracted from deep-seated lymphoid organs (the spleen and the bone marrow). We expect a skewed immunological balance towards an immunoregulatory phenotype (Th1), as well as modulation of RA-induced chronic inflammation. In another approach, CIA-HIS mice may receive co-therapy treatment followed by adoptive transfer of NKB cells (**Figure 2B**). Immune homeostasis may be analyzed to determine whether the immunological balance is tipped towards the Th1 cytokine expression profile. These proposed future studies aim to confirm whether NKB cell expansion is inhibited in the lymphoid organs of CIA-HIS mice receiving co-therapy (**Figure 2B**) in order to maintain the immunoregulatory phenotype (48–51).

The low expression of CD40 during stimulation fails to activate Akt (a downstream effector of PI3K-Akt signaling), thus limiting the translocation of NF-κB from the cytoplasm to the nucleus due to its intact inhibitor IκB. We believe that human THP-1 macrophages (phorbol myristate acetate (PMA)-differentiated human THP-1 monocytes) receiving co-therapy treatment are regulated by the FOXO1 transcription factor. We also believe that they mediate the pro-apoptotic protein Bim expression, which drives programmed death of LPS-stimulated human macrophages (**Figure 2C**).

CIA-HIS mice may prove to be a viable preclinical tool to confirm whether co-therapy can suppress the inflammation-dependent conversion of Th1 to Th17 cells (**Figures 2A, B**). The CIA-HIS mice that receive co-therapy may exhibit reduced inflammation controlled by expanding NKB cells, inducing programmed death of pathogenic RA cells. The maintenance of an immunoregulatory phenotype by controlling the conversion of Th1 to Th17 cells, using our proposed co-therapy regimen in CIA-HIS mice, should provide a better understanding of the role played by NKB cells during RA.

## Conclusions

The proposed novel immune cell lineage, namely, NKB cells, is of significant interest in the development of therapeutic interventions for infectious and inflammatory diseases. The protective role played by NKB cells during infectious diseases, and their role in maintaining immune homeostasis by promoting an immunoregulatory environment, suggests that they can critically contribute to health and disease. The maintenance of an immunoregulatory (Th1), rather than a pathogenic (Th17), immune cell phenotype in HIS mice may provide an important impetus to develop therapeutic interventions for systemic inflammatory diseases. Finally, the interactions of NKB cells with other immune cells may be further explored to expand the current knowledge on host-pathogen interactions.

## Author contributions

Conceptualization: NT and RKT; resources and information collection: NT; writing—original draft preparation: NT, SN, and RKT; writing—review and editing: NT and RKT. All authors contributed to the article and approved the submitted version.

## Funding

RKT would like to express his gratitude to the Ramalingaswami Re-entry Fellowship Project, DBT, New Delhi (No. BT/RLF/Re-entry/27/2018), the Indian Council of Medical Research (ICMR), New Delhi extramural grant (35/1/2020-Nano/BMS) for generously supporting this study. NT would like to thank the Indian Council of Medical Research (ICMR) for providing the fellowship to carry out his research (ICMR award letter No.: 2020-7623/CMB-BMS).

## Acknowledgments

RKT would like to express his thanks to the central MIL facility of CSIR-IMTECH, Chandigarh. NT would like to thank Nirma University for facilitating this research work.

## Conflict of interest

The authors declare that the research was conducted in the absence of any commercial or financial relationships that could be construed as a potential conflict of interest.

## Publisher’s note

All claims expressed in this article are solely those of the authors and do not necessarily represent those of their affiliated organizations, or those of the publisher, the editors and the reviewers. Any product that may be evaluated in this article, or claim that may be made by its manufacturer, is not guaranteed or endorsed by the publisher.

## Abbreviations

ACE, Aceclofenac; ALD, Alcoholic liver disease; AsC, Asymptomatic HBV carrier; BCR, B-Cell receptor; BMMSCs, Bone marrow derived mesenchymal stem cell; CDR3, Complementarity-determining region 3; CHB, Chronic hepatitis B; CIA, Collagen induced arthritis; GSF, Gingival crevicular fluids; HBV, Hepatitis B virus; HBV-ACLF, HBV-associated acute-on-chronic liver failure; HC, Healthy control; HIS, Humanized immune system; IFN-γ, Interferon-γ; ILCs, Innate lymphocyte cells; IL-18BP, IL-18 binding protein; LN, Lymph nodes; LPS, Lipopolysaccharide; LPHNPs, Lipid-polymer hybrid nanoparticles; MLNs, Mesenteric lymph nodes; MTX, Methotrexate; MZ, Marginal Zone; NK cells, Natural killer cells; NKB, Natural killer-like B cells; NKBP, NKB precursor; NLCs, Nanostructure lipid carriers; PBMCs, Peripheral blood mononuclear cells; RA, Rheumatoid arthritis; SIV, Simian immunodeficiency virus.
